# Editorial: Advancements and prospects of genome-wide association studies

**DOI:** 10.3389/fgene.2025.1564006

**Published:** 2025-02-19

**Authors:** Ayo P. Doumatey, Yafang Li, Juan Carlos Fernandez-Lopez

**Affiliations:** ^1^ Center for Research on Genomics and Global Health, National Human Genome Research Institute, National Institutes of Health, Bethesda, MD, United States; ^2^ University of New Mexico Comprehensive Cancer Center, Albuquerque, NM, United States; ^3^ The National Institute of Genomic Medicine (INMEGEN), Mexico City, Mexico

**Keywords:** translation, eQTL, LDL - cholesterol, mendelian randomization, asthma, transferability, omics integration

Since the very first Genome-Wide Association Study was published in 2005 followed by the pivotal manuscript by the Wellcome Trust Case Control Consortium (WTCCC) in 2007 (Klein et al., 2005; Wellcome Trust Case Control, 2007), several thousands of Genome-Wide Association Studies (GWAS) have been conducted (>45,000) and yielded a myriad of loci associated with diverse human phenotypic traits or diseases (>5,000) (Sollis et al., 2023). These studies have not only shed light on the involvement of common variants but have also discovered important insights into biology (Uffelmann et al., 2021).

The field of GWAS has seen tremendous advances both technologically and methodologically. For example, GWAS have been facilitated by single nucleotide polymorphisms (SNPs)-arrays containing a couple of hundred thousand to 2 million SNPs (Visscher et al., 2017). The ability to impute untyped SNPs has increased marker density, improved the statistical power of GWAS, and enabled large-scale meta-analyses across populations, but also aided in fine-mapping regions of interest in genetic loci associated with diseases or phenotypic traits (Cahoon et al., 2024; Zhihui Zhang et al., 2022). Additionally, with the advances in sequencing technologies and the drop in sequencing cost, the latest GWAS and follow-up studies have been sequence-based and allowed for the assessment of low-frequency and rare variants in association studies (Acar et al., 2023; McMahon et al., 2021; Pan et al., 2023).

Despite all the advances in Genomics, not all global populations are evenly represented in GWAS. GWAS have been mainly limited to populations of European descent and could exacerbate existing health disparities and the implementation of precision medicine (Doumatey et al., 2023). The push for genomic evenhandedness and representation of all global populations in biomedical research has led to initiatives such as the 1000 Genomes Project, the Human Heredity& Health (H3Africa), the Trans-omics for Precision Medicine (TOPMed), and the All of Us in recent years (Auton et al., 2015; Investigators, 2024; Peprah et al., 2017; Taliun et al., 2021). Some of these studies have improved imputation references, mainly 1,000 genomes and TOPMed (Kowalski et al., 2019). Others, such as H3Africa, have helped develop population-specific genotype arrays, thus improving discovery in these populations. In this Research Topic, Brandenburg et al. used such custom array enriched for African common variants, the H3A custom genotyping array, in the largest GWAS for urinary albumin-to-creatinine ratio (UACR), a biomarker of kidney disease, in Sub Saharan Africans living in different regions of the continent and non-resident individuals of African ancestry (United Kingdom BioBank) and African American cohorts (CKD-Gen African Ancestry). The authors identified two novel variants associated with UACR in populations of African ancestry (BMP6, HBB), and they replicated only three out of 60 known UACR-associated loci previously identified in European and multi-ancestry studies. Additionally, the authors performed polygenic score (PGS) and comparison analyses to evaluate the transferability of PGS derived from non-African and multi-ancestry populations to African populations.

A comprehensive review by Bruner and Grant in this Research Topic underscored the need to account for population-biased findings by improving representation across various ancestral populations, which should, in turn, address replication and transferability challenges across populations. The authors especially highlighted advancements in methods and techniques and available resources to conduct functional annotations of GWAS variants and mechanistic “variant-to-function” follow-up studies. They described functional annotations (e.g., epigenetic annotations, pathway and network analyses, quantitative trait loci) and functional validations (reporter assays, genome editing, and animal models) as approaches to characterize identified GWAS variants and associated effector genes.

Some of these methods were used in the studies included in this Research Topic. In studies conducted by Abbas et al. and Brandenburg et al. in populations of African ancestries, the authors leveraged expression quantitative trait locus (eQTL) analyses to understand the mechanisms connecting identified GWAS variants to phenotypes (ref. Abbas et al.; Brandenburg et al.) using two different strategies. Brandenburg et al. first conducted a GWAS and subsequently carried out a functional annotation of the lead single nucleotide polymorphisms (SNP) associated with UACR using relevant tissue expression profile (glomerular and tubulointerstitial tissues) for a cis-eQTL analysis. In contrast, Abbas et al. first conducted a differential expression analysis to identify mRNAs that were differentially expressed between Low-Density Lipoproteins (LDL) groups (low tertile vs. upper tertile), then extracted all SNP in the cis region of each mRNA differentially expressed between LDL groups to perform an eQTL analysis. eQTLs significantly associated with differentially expressed mRNAs were overlapped with genetic variants previously associated with LDL and LDL traits in the GWAS catalogue. The authors also used pathway and network analyses to further decipher functional relationships between transcriptomics, genomics, and phenotype. Noteworthy, an African-specific eQTL was associated with TTC38 expression in African Americans. TTC38 was significantly upregulated in individuals with elevated LDL.

Animal models are proposed as a variant-to-function method to understand GWAS discovery. These methods can take many years but effectively decipher the underpinnings of biology in humans. In one of the studies, Fowler et al. conducted a GWAS in heterogeneous stock (HS) to identify genomic regions associated with intraocular pressure (IOP). The authors indicated a few advantages for conducting GWAS and follow-up studies in rats for phenotypes like IOP: 1) the ability to control the environmental variables known to affect IOP in humans, 2) rats and humans share similar anatomical and developmental characteristics of the eyes (e.g., the aqueous outflow pathway), thus an ideal model to study pathophysiology of ophthalmologic disorders, 3) tissues for phenotyping and gene expression can be easily collected. The combination of GWAS in ∼1800 HS rats identified 5 candidate genes, of which two were novel (Ctsc2 and Plekhf2-never reported in human studies); the authors concluded that the use of HS rats resulted in new findings that could provide new insights into the molecular basis of IOP.

GWAS can identify genetic variants linked to a trait/disease, but they cannot always establish causality. Thus, epidemiology-based methods such as Mendelian Randomization (MR) have been used to establish causality between genetic variants, used as instrumental variables, risk factors and lifestyle factors of several diseases (Benn and Nordestgaard, 2018). Lin et al. used this strategy to establish a causal relationship between several reproductive factors (e.g., age at menopause, age at menarche, age at first live birth) and bone density. Their findings suggest that early menopause and late childbirth may be important predictive biomarkers of bone density decrease.

Similarly, Li et al. used both univariate MR and multivariate MR to evaluate causality between childhood-onset asthma, adult-onset asthma, and the risk of abnormal spermatozoa. As with many MR studies, this study leveraged large-scale GWAS meta-analysis conducted in well-known biobanks and studies such as the United Kingdom bank and the FinnGen study to obtain summary statistics for the risk factors included in the MR and the instrumental variable.

The six articles published in this Research Topic summarized well the current advancements and prospects in genome-wide association studies. Notably, the discovery GWAS in diverse populations, the inclusion of multiple -omics in study designs and the development of new methods to efficiently integrate these omics for GWAS finding follow-ups, the leverage of existing large biobanks, and animal models for variant-to-function studies remain critical for the future of genomics. Even though artificial intelligence (AI) was not explicitly used in the original research papers published in this Research Topic, AI-based methods have been integrated into GWAS and follow-up study pipelines. For example, AI integration in data management or tools that infer the functional impact of a variant (e.g., GeneMANIA, PhenoScanner, STRING, Escape) (Bruner and Grant).

## References

[B1] Acar I. E. Galesloot T. E. Luhmann U. F. O. Fauser S. Gayán J. den Hollander A. I. (2023). Whole genome sequencing identifies novel common and low-frequency variants associated with age-related macular degeneration. Invest Ophthalmol. Vis. Sci. 64 (14), 24. 10.1167/iovs.64.14.24 PMC10664724 37975850

[B2] Auton A. Brooks L. D. Durbin R. M. Garrison E. P. Kang H. M. Korbel J. O. (2015). A global reference for human genetic variation. Nature 526 (7571), 68–74. 10.1038/nature15393 26432245 PMC4750478

[B3] Benn M. Nordestgaard B. G. (2018). From genome-wide association studies to Mendelian randomization: novel opportunities for understanding cardiovascular disease causality, pathogenesis, prevention, and treatment. Cardiovasc Res. 114 (9), 1192–1208. 10.1093/cvr/cvy045 29471399

[B4] Cahoon J. L. Rui X. Tang E. Simons C. Langie J. Chen M. (2024). Imputation accuracy across global human populations. Am. J. Hum. Genet. 111 (5), 979–989. 10.1016/j.ajhg.2024.03.011 38604166 PMC11080279

[B18] Doumatey A. P. Bentley A. R. Akinyemi R. Olanrewaju T. O. Adeyemo A. Rotimi C. (2023). Genes, environment, and African ancestry in cardiometabolic disorders. Trends Endocrinol. Metab. 34 (10), 601–621. 10.1016/j.tem.2023.07.007 37598069 PMC10548552

[B5] Investigators A. o. U. R. P. G. (2024). Genomic data in the all of us research Program. Nature 627 (8003), 340–346. 10.1038/s41586-023-06957-x 38374255 PMC10937371

[B6] Klein R. J. Zeiss C. Chew E. Y. Tsai J. Y. Sackler R. S. Haynes C. (2005). Complement factor H polymorphism in age-related macular degeneration. Science 308 (5720), 385–389. 10.1126/science.1109557 15761122 PMC1512523

[B7] Kowalski M. H. Qian H. Hou Z. Rosen J. D. Tapia A. L. Shan Y. (2019). Use of >100,000 NHLBI Trans-Omics for Precision Medicine (TOPMed) Consortium whole genome sequences improves imputation quality and detection of rare variant associations in admixed African and Hispanic/Latino populations. PLoS Genet. 15 (12), e1008500. 10.1371/journal.pgen.1008500 31869403 PMC6953885

[B8] McMahon A. Lewis E. Buniello A. Cerezo M. Hall P. Sollis E. (2021). Sequencing-based genome-wide association studies reporting standards. Cell Genom 1 (1), 100005. 10.1016/j.xgen.2021.100005 34870259 PMC8637874

[B9] Pan H. Liu Z. Ma J. Li Y. Zhao Y. Zhou X. (2023). Genome-wide association study using whole-genome sequencing identifies risk loci for Parkinson's disease in Chinese population. NPJ Park. Dis. 9 (1), 22. 10.1038/s41531-023-00456-6 PMC9911746 36759515

[B10] Peprah E. Wiley K. Sampson U. Narula J. (2017). A new age for african-driven genomics research: human heredity and health in africa (H3Africa). Glob. Heart 12 (2), 67–68. 10.1016/j.gheart.2017.05.003 28867289

[B11] Sollis E. Mosaku A. Abid A. Buniello A. Cerezo M. Gil L. (2023). The NHGRI-EBI GWAS Catalog: knowledgebase and deposition resource. Nucleic Acids Res. 51 (D1), D977–d985. 10.1093/nar/gkac1010 36350656 PMC9825413

[B12] Taliun D. Harris D. N. Kessler M. D. Carlson J. Szpiech Z. A. Torres R. (2021). Sequencing of 53,831 diverse genomes from the NHLBI TOPMed Program. Nature 590 (7845), 290–299. 10.1038/s41586-021-03205-y 33568819 PMC7875770

[B13] Uffelmann E. Huang Q. Q. Munung N. S. de Vries J. Okada Y. Martin A. R. (2021). Genome-wide association studies. Nat. Rev. Methods Prim. 1 (59), 59. 10.1038/s43586-021-00056-9

[B14] Visscher P. M. Wray N. R. Zhang Q. Sklar P. McCarthy M. I. Brown M. A. (2017). 10 Years of GWAS discovery: biology, function, and translation. Am. J. Hum. Genet. 101 (1), 5–22. 10.1016/j.ajhg.2017.06.005 28686856 PMC5501872

[B15] Wellcome Trust Case Control C. (2007). Genome-wide association study of 14,000 cases of seven common diseases and 3,000 shared controls. Nature 447 (7145), 661–678. 10.1038/nature05911 17554300 PMC2719288

[B16] Zhihui Zhang X. X. Wen Z. Zhu D. Amos C. I. (2022). False positive findings during genome-wide association studies with imputation: influence of allele frequency and imputation accuracy. Hum. Mol. Genet. 31 (1), 146–155. 10.1093/hmg/ddab203 PMC8682785 34368847

